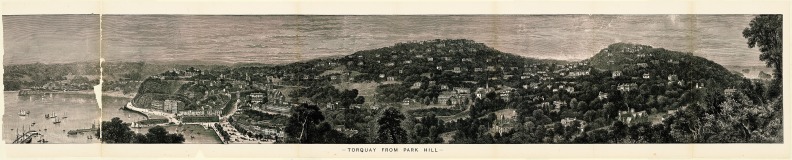# Health-Resorts in the West of England and South Wales. Torquay

**Published:** 1893-06

**Authors:** Paul Q. Karkeek

**Affiliations:** Medical Officer of Health for Torquay and St. Marychurch


					^ealtMResorts in tbe West of J6nglan&
anb Soutb Wlales*
VIII.
TORQUAY.
BY
Paul Q. Karkeek, M.R.C.S. Eng., L.S.A.,
Medical Officer of Health for Torquay and St. Marychurch.
here are very few places in England where such a delightful
Vlew is obtained immediately on leaving the railway station as
!n ^0rciuay. In but too many instances, the entrance to a town
? through some unpleasant-looking slum or third-class suburb.
re' ?n the contrary, the view on approaching the town is one
^nest possible. Numerous villas, situated on the slopes
0 hills or on the edge almost of steep cliffs, interspersed with
c v clumps of evergreen foliage, look down on the waters of
. r ay, which seem to approach as if to kiss the feet of Torquay
a11 her beauty. The tout ensemble makes a most attractive
94 HEALTH-RESORTS.
picture, and produces an excellent and inspiriting effect on the
traveller reaching the town, wearied perhaps by a long journey.
And yet a hundred years ago barely a germ of the Torquay
of to-day was to be seen. A miserable little pier or breakwater
to shelter the fishing boats, a small boat-building yard, a mill,
and a few?very few?cottages scattered about was all that then
existed. It was called Tor Quay to distinguish it from Tor, or
Old Tor, or Torre, where was the parish church, and which was
the best part of a mile away. In those days the people at Tor
spoke of the sea-board portion of the parish as " Down at the
Quay," and doubtless, like other old inhabitants, looked down
with no small contempt on the Quay folk.
The story of Tenterden steeple being the cause of the
Goodwin Sands is well known; but almost as curious is the
origin of the growth of Torquay, inasmuch as without exaggera-
tion it may be distinctly ascribed to the French Revolution-
During the wars which preceded and accompanied this volcanic
irruption of the human race, fleet after fleet assembled in
Torbay, and sometimes a ship would be waiting here for months
until the tale of her consorts was complete, and they could be
sent away together on their mission of destruction. When an
officer was ordered to take his ship to Torbay and wait for further
orders, what more natural than that he should communicate
with his family, and that his wife and children should come down
into Devonshire and stay somewhere near the ship until she
sailed ? This was constantly going on, more or less, from
about 1780 until 1815, and to suit the requirements of these
visitors a few cottages were built, and then a few rows oi
houses ; but all of a small and inexpensive character. During
these visitations, it was sometimes observed that one or other
member of these families who seemed in a bad way from lung
diseases when brought down here, managed somehow to outlive
or outgrow this delicacy in an unwonted manner. Consumption
and its allies influence the destinies of only too many families,
and consequently it did not take long to establish a reputation
for Torquay, a place which has since grown from the little fishing
village -to the beautiful town which we now see. In the year
1801 the population was 838, living in 143 houses; when
TORQUAY. 95
?Napoleon, a prisoner on board the Bellerophon, looked on the
sP?t there were less than 300 houses, with about 1,500 inhabi-
tants. From this time the increase every ten years was by
leaPs and bounds. In 1821 the number was 1,925; in 1831,
3>58o; in 1841, 5,980; in 1851, 11,470; in 1861, 16,400; and
"when, in 1871, the nephew of the first Napoleon, the so-called
?Man of Sedan, came here an exile in his turn, the population
had grown to 21,657, and the number of inhabited houses
lncreased to 3,071. At the last census, in 1891, it was found
that the population was 25,500, and the number of inhabited
houses 4,470. Very little of Torquay proper remains on
^hich to build, but her sister parishes, St. Marychurch and
C?ckington, prospering on her renown, have not oeen idle;
^deed, the sum total of population of the Torquay postal
^strict is not far short of 35,000.
There is not in all Torquay a level piece of ground large
enough for a cricket-field; it consists entirely of hills and their
valleys, and some of these hills rise to a height of 450 feet
above sea level. There is really but one street, although it
hears different names in its course of over a mile. Starting as
Norwood Street, it soon becomes The Strand, and then Fleet
Street; then Lower Union Street, and lastly Higher Union
Street. There are some fine terraces, but the style of residence
ni?st affected is the villa; and this can be seen here in every
Possible shape and size, from the cottage to the mansion. Each
vUla is surrounded by a garden, and the boundaries of each
Seeni lost in plantations of evergreen shrubs, which grow here
Vvith great luxuriance. For the most part, the foundations are
011 solid limestone rock, which assures a dry subsoil; and for
th
le same reason, the roads soon dry, thus enabling the delicate
*? go out of doors directly after it has ceased raining. The total
rainfall in 1892 was 24.13 inches, falling on 145 days, but on 23
*here was a fall of only 0.01 : it is, however, sometimes higher
than this.
The peculiarity of Torquay is the equability of its climate,
^hich certainly is very marked. During 1892 the mean of
*he maximum temperature was 55.8, and the mean of the
Minimum temperature was 44.4. The mean of the maximum
g6
HEALTH-RESORTS.
and minimum together was 50.1, and the mean range of tempera-
ture 11.4, or exactly the same as at Madeira in 1892. It will
thus be seen that the tremendous range so distressing to those
who frequent the South of France is unknown here. Too great
stress cannot be laid on this fact; for although it may be very
delightful to be in the hot sunshine all day, it is just as dis-
tressing to have intensely cold nights. For those with a feeble
circulation, there can be no greater trial of endurance than to
keep warm at night under adverse circumstances ; and artificial
heat, no matter how distributed, is at the best but a poor
substitute for the natural supply.
Doctors differ, and so do those learned in climatology; but
the fact remains, there is in the climate and the weather that
which no instrument has yet been able to record. Call it what
one may, there is a something in the air of Torquay which
enables a patient with a raw lung to breathe with ease, and
another with a winter cough to escape that distressing annuity-
Nature goes on her own way, and shows in a manner peculiarly
her own what she thinks on such matters; and as I write this*
in the month of February, there can be seen in one of the public
gardens an orange tree with green and yellow fruit, which
has been planted there for two or three years. Close by *s
a red camellia in full bloom, and a few feet farther on is a
magnolia with numerous white flowers fully out. The eucalyptus
does well in any sheltered spot, and so do the dracaena, the
true palm, hill and swamp flax, and hill bamboo ; the yucca
and aloe frequently flower here. When it is remembered that
nearly all the town has a south and south-west aspect, and that
enormous hills act as a wall against the east and north-east, it
will be easily understood that it is not difficult to gather in each
garden a collection of plants such as it would be almost impos-
sible to keep alive in any other part of England. Some of the
residents are fond of making experiments in their gardens, and
those of Bemerton, on the Warren Hill, and Duncan House,
Torwood, must be seen to be understood.
There is another curious fact about the climate of Torquay '>
viz., the coolness of the summer. It is a popular delusion th&t
because a place is mild in winter, that therefore it is very
-TORQUAY FROM PARK HILL
-TORQUAY FROM PARK HILL
TORQUAY. g7
h?t in summer. The spot of land on which Torquay is built
s almost an island. Torbay encroaches on one side and
bacombe Bay on the other, and the proximity of the sea
r^nders the air cool. Now, as there are no white cliffs to reflect
e sun's rays, but only dark green foliage in the background, it
. ?Ws that no matter how hot the sun is elsewhere, in Torquay
ls always cool; indeed, it is a rare occurrence for the thermo-
meter in the shade to reach 70?. Here again Nature bears
e;idence of a very striking character: those who attempt to
&r?\v peaches out of doors may perhaps get a crop to ripen
?ut once in five or six years ; while at Exmouth, only a few
miIes away, no difficulty whatever is found.
Without going so far as to say that the climate of Torquay
Slntable for every person, or for every kind of ailment, it may
eWorthwhile to point out what class of cases generally do well
here.
AH patients requiring rest and quiet in a mild and soothing
ftiate improve greatly. If brought early enough, delicate
. 1 ren will outgrow their weakness, and the troubles of puberty
?Jrls are for the most part easily overcome. Often enough
blris are sent here almost bloodless from excessive menstruation :
first they are not infrequently surprised at a total cessation,
lch may last a few months ; and then, with restored strength,
?rmal flow is established. The many forms of tubercular
^1S?hief in children, especially in the early stages, answer to
^atrrient here. Those curses of old age, the winter cough and
k ?nic bronchitis, are reduced to a minimum ; and there can
n? doubt that hundreds of elderly people manage to live
n for years in Torquay whose days seemed only too plainly
?j, bered when in their own homes in the North. In fact,
^uay is preservative to old age.
clj *s n?t the place to discuss the vexed question of the
tij^^0 *reatment of consumption; opinions have varied from
pose *? time, and will continue to do so until the end of time, sup-
rerJn^ ^ere be such a disease in those days; but the following
fa' a^S are result of long observation, and written in all
jjj ^GSS' The class of cases which do best are those sent here
earliest stages?those with bronchial catarrh and irrita-
98
HEALTH-RESORTS.
bility of the mucous membrane. Next in order as likely to
derive benefit are those in the later stages, provided the mischief
is not active; the senile forms often do nicely, and cases com-
plicated with asthma frequently obtain considerable alleviation-
The rapidly advancing and advanced cases, with large excava-
tions, much suppuration, hectic and rapid wasting, are no more
likely to do well here than elsewhere; although most of the
Torquay practitioners can tell of extraordinary cases which)
apparently hopeless, have yet greatly improved. And here it
must be noted that every year patients are sent here who ought
never to have left their home comforts and surroundings, nor
to have been submitted to the fatigue and exposure incidental
to transit.
Cases of irritable nervous affections, with deficient sleeping
power, generally do well. Overworked brains derive great
benefit from a stay here, provided rest, quiet, and regularity
diet are secured. Acute rheumatism is very rare, and many
forms of chronic rheumatism get on well. Bright's disease is not
a common ailment, calculous affections are extremely rare, and
those who live according to the requirements of the climate at6
seldom troubled with liver. Those, on the other hand, who eat
lots of cream and other rich things, and drink as if they were &
some cold and bracing climate, of course soon come to grief'
and thus learn by experience that in Rome they should do &
the Romans do. Most cases of non-neurotic constipation
well without any treatment whatever; the mild air and the soft
drinking-water seem to act on these cases like a charm.
The water supply of the town is collected from Moorlan^
streams on one of the offshoots of Dartmoor, about seventeen
miles away. There are two reservoirs, holding together abo^
three hundred millions of gallons. The constant service is ^
vogue, and last year, after the longest drought on record, thefe
were still seventy millions of gallons in store, or about three
months' supply. The water is very pure and remarkably soft'
and cases of lead poisoning are unknown.
The main drainage was completed some years ago at a cO^
of over ^"80,000, under the superintendence of the late ^
Joseph Bazalgette, and is a very perfect and satisfactory wo^
TORQUAY. 99
all respects. For many years efforts have been made to
Secure the general health of the town by attention to the indi-
^dual house, and excellent results have resulted therefrom.
6re 1S no reSister certified houses kept, because it is felt
a there is nothing made by man which will keep in order for
ever? i i ? .
> and consequently a house that may be in good condition
year and certified for accordingly, may be very defective
next. Any intending occupant can have a house examined
^ y a borough official free of charge, who will report on the con-
>?n of the house in question, and point out defects if any
Xlst- Visitors are strongly advised to ask for " a fresh sanitary
Certificate from the Town "Hall." That this plan has not been
?rked jn vajn bg seen from the following table:
Causes of Death in Torquay for Ten Years.
CAUSES.
Small pox
'o;a?atina ??? ?"
^ Phtheria... ^
' ;;;
?nteric or Typhoid ...
Continued ...
lapsing
^siPeias;:; "*
\v?s1k ::: ::: :::
Diar Cough
RhPir oea and Dysentery
Fever ... '
>isis
''lell"sy. anil
?srs!onia - ?? ??
SH- *::: ::: :::
?ther Diseases
'83
56
76
3?
5
211
?84 '85
3
1
15
o
o
4i
64
32
5
218
60
218
'86 '87
4M 385 1396 I412 I333
4
3
3
5
o
61
59
39
3
228
52
38
39
9
184
'88
90
1
3
o
64
52
39
6
228
10
199
16
67
63
36
8
235
370 I429 (402 I420
'91 | 92
o
9
4
o
67
53
44
9
214
60
64
38
13
228
Th
but GSe ^Sures an average mortality of 15.5 per 1,000;
str every year at least fifty deaths are entered as visitors,
*3.nor0?._
etc ^ sailors, tramps, patients sent to the various hospitals,
bg ' etC"' an^ if these be deducted the average will be found to
^eath'^ 1,000' surely a very low figure. The zymotic
rate for the ten years is .6 per 1,000.
100 PROGRESS OF THE MEDICAL SCIENCES.
Compulsory notification is in force, and there is a well-
arranged hospital for isolating infectious diseases, where private
rooms can be obtained.
For visitors of a scientific turn of mind the museum of the
Natural History Society offers considerable attraction. Here
will be found specimens of Devonshire fauna and geology, and
the wonderful finds resulting from the exploration of Kent's
Cavern. For those inclined to sport, there are fishing, cricket,,
tennis, boating and yachting, and one of the finest golf links in
the kingdom. In addition to the usual attractions of seaside
resorts, such as concerts, theatres, and reading-room, lending
libraries, and so on, there is a series of walks and drives within
easy reach of surpassing beauty. Excursions to the various
parts of Dartmoor can easily be arranged, and the coast line
for many miles offers at every turn some special beauty and
charm.
It is no easy matter to describe in adequate terms a country
so beautiful as this, there are so many sides from which to
enjoy it: whether it be the naturalist, the artist, or the dreamy
poet, each in his own way will express the delight he finds; but
probably all will agree with Mr. Ruskin, and call it " The Italy
of England."

				

## Figures and Tables

**Figure f1:**